# Proteomic analyses in early brain development and neuropathological implications of fetal growth restriction

**DOI:** 10.4103/NRR.NRR-D-25-01234

**Published:** 2026-01-27

**Authors:** Gemma C. Ventura, Kirat K. Chand, Paul B. Colditz, Julie A. Wixey

**Affiliations:** 1 UQ Centre for Clinical Research, Faculty of Medicine, The University of Queensland, Brisbane, QLD, Australia; 2 Perinatal Research Centre, Royal Brisbane and Women’s Hospital, Brisbane, QLD, Australia

**Keywords:** biomarker, brain development, fetal growth restriction, neuropathology, proteomics

## Abstract

Proteins are the primary functional units within cells, driving complex biological processes essential for stem cell differentiation into specific neural lineages and for the structural and functional maturation of the central nervous system. Advances in high-throughput proteomic technologies allow comprehensive profiling of molecular landscape of the brain, revealing dynamic, region- and time-specific changes in protein expression. During early embryonic development, pluripotency-associated proteins are highly expressed but gradually decline as lineage-specific markers and pathways governing DNA regulation and cytoskeletal organization become predominant. In fetal and postnatal stages, synaptic and metabolic proteins are enriched in a region-specific manner, reflecting functional compartmentalization and specialization in the central nervous system. Fetal growth restriction is an obstetric complication caused by sustained periods of inadequate oxygen and nutrient supply, preventing the fetus from achieving its genetic growth potential. Proteomic analysis of fetal tissues and biofluids has deepened our understanding of the molecular mechanisms associated with fetal growth restriction, highlighting metabolic and vascular adaptations, inflammatory responses and redox imbalances. These analyses have also uncovered molecular signatures with potential value as biomarkers for clinical diagnosis (e.g., complement proteins in maternal blood in fetal growth restriction), and prognosis (e.g., neurogenic locus notch homolog protein 1 as a modulator of fetal growth restriction response). Despite such advances, animal models remain indispensable for elucidating the multifactorial nature of fetal growth restriction-related neuropathology, pinpointing region-specific alterations. They also offer a controlled setting to explore how factors such as sex, gestational age at birth, and birth weight influence the impact of fetal growth restriction on brain development. A deeper understanding of neurodevelopmental processes and the pathological mechanisms involved in fetal growth restriction is critical for the development of effective diagnostic strategies and targeted therapeutic interventions. The purpose of this review is to provide synthesis of neuroproteomic alterations across developmental stages, highlighting how chronic intrauterine oxygen and nutrient deprivation, in humans and animals, shapes the proteomic landscape.

## Introduction

Brain development is a complex and dynamic process that begins in utero and continues into adulthood, progressing through tightly regulated stages. The biological mechanisms involved are dependent on gene expression, which drives the production of protein molecules essential for neural stem cell (NSC) differentiation, migration and maturation (Negi and Guda, 2017). These molecular processes ultimately guarantee the structural and functional compartmentalization of the structures that make up the central nervous system (CNS) (Stiles and Jernigan, 2010). Proteins are the primary effectors of cellular function, performing a wide range of biological processes to maintain cellular homeostasis. Their expression is tightly regulated across different cell types, brain regions, and developmental stages, adjusting in response to intrinsic genetic programs and environmental demands (Moaddel et al., 2021).

Early neuroscience research focused on single or small sets of proteins, which could not capture the dynamic interactions and network formations that translate genetic programs into functional outcomes. The emergence of high-throughput proteomics has transformed this landscape, enabling simultaneous quantification of thousands of proteins and, linking molecular cascades to cellular phenotypes and neurodevelopmental trajectories (Basu et al., 2021). These advances can be leveraged to identify the molecular drivers underlying neuropathological conditions.

Two-dimensional gel electrophoresis was the first method applied for the analysis of protein profiles, allowing the separation of intact proteins based on molecular weight and isoelectric point (Lee et al., 2020). Although two-dimensional gel electrophoresis remains a valuable tool to identify protein isoforms and posttranslational modifications (PTM) (Lee et al., 2020), mass spectrometry (MS)-based techniques have now become the gold standard for proteomic analysis, offering superior sensitivity and accuracy by identifying protein molecules based on their mass-to-charge ratio (m/z) and quantifying their abundance through signal intensities (Dai and Shen, 2022). Moreover, advances in complex software tools have further expanded proteome coverage and sensitivity, enhancing the analytical power of proteomics. Data independent acquisition (DIA) techniques have become central for comprehensive proteome profiling, fragmenting all ions within defined m/z widows. Strategies such as XDIA, SWATH-MS, or BoxCar DIA enable precise detection of subtle temporal and region-specific proteomic changes (Kitata et al., 2023), making them suitable for studying the dynamic neurodevelopmental proteome.

These technological and computational advances have been reinforced by global efforts to standardize proteomic research, ensuring reproducibility, comparability, and the generation of reference datasets. Within this framework, the Human Proteome Organization was established in 2001 to unify global efforts in proteomics research (Abbott, 2001). Under this initiative, the Human Proteome Organization Brain Proteome Project was introduced to set standardized guidelines for brain-specific proteomic analyses (Hamacher et al., 2006). Large-scale proteomic analyses conducted under these standards have become instrumental in elucidating molecular mechanisms underlying both physiological and pathological brain development, with specific protein profiles increasingly recognized as potential biomarkers for diagnosis, prognosis and therapeutic targeting (Johnson et al., 2006).

The developing brain is particularly metabolically active, consuming 60% of the total body oxygen and 40% of glucose during critical growth windows. As such, adequate oxygen and nutrient supply are essential for physiological brain growth (Cacciatore et al., 2022). Fetal growth restriction (FGR) is one of the major causes of perinatal morbidity and mortality. It results from prolonged deprivation of oxygen and nutrients, often due to placental insufficiency, which hinders the fetus from achieving its genetic growth potential (Armengaud et al., 2021; Dudink et al., 2022). FGR not only impairs growth but also poses a significant risk to adverse neurodevelopmental outcomes, including delays in motor function, cognitive impairments, and behavioral disturbances. These outcomes are influenced by factors such as the severity of placental dysfunction, time of onset, gestational age at birth and birth weight (Armengaud et al., 2021; Dudink et al., 2022). Importantly, adaptive response of the brain to chronic hypoxia and hypoglycemia is reflected in protein profiles, which can be leveraged to gain insights into the biological mechanisms causing brain injury (Johnson et al., 2006). While human analyses have shed light on how proteomic profiles evolve during brain development, they often focus on discrete time points and a narrow selection of brain regions, limiting our understanding of longitudinal and region-specific changes. In pathological conditions, analyzing body fluids for proteomic alterations can aid in disease characterization and biomarker discovery, but such approaches often fail to link molecular changes with neurological outcomes and the specific brain areas involved. Animal analyses are uniquely positioned to overcome these limitations, enabling region-specific, longitudinal investigations under controlled experimental conditions.

This review aims to provide an integrated and critical synthesis of neuroproteomic changes across developmental stages, emphasizing how chronic intrauterine oxygen and nutrient deprivation shape the proteomic landscape in both humans and animal models.

## Search Strategy

This review covers proteomic studies on brain development and FGR pathology published from 2006 to the present. Literature was retrieved from PubMed and Web of Science, and complemented by Google Scholar searches. The search strategy combined the following key terms: proteomics, brain, embryonic development, fetal development, postnatal development, brain organoids, FGR, body fluids, fetal compartments, and animal models.

## Evolution of Proteomic Profiles during Early Neurodevelopmental Stages

### Embryonic development

The development of the human brain begins in the third week of gestation with a process known as neurulation. During this phase, ectodermal cells receive molecular signals from the notochord, prompting them to form the neural tube, which will eventually develop into the CNS (Stiles and Jernigan, 2010). A substantial amount of research has been dedicated to the molecular profiling of differentiating human embryonic stem cells (hESCs) into neural cells, offering valuable insights into human neurogenesis and presenting potential therapeutic applications.

Early analyses in this field support the dynamic nature of the proteome during neural differentiation. The downregulation of the peroxidase 4, involved in the nuclear factor-kappa beta anti-apoptotic pathway, along with a decrease in protein linking integrin associated protein to cytoskeleton 1, a protein known to protect neurons against apoptosis, was thought to regulate the pro-apoptotic signaling during differentiation. Other proteins differentially regulated were associated with nucleic acid functions, highlighting the role proliferating cell nuclear antigen as a marker of proliferating cells, alongside other differentially regulated cell cycle-related proteins. Other 45 proteins involved in cytoskeletal organization, a crucial mechanism for cell division and proliferation, were differential expression (DEX) (Hoffrogge et al., 2006). The upregulation of cytoskeleton-associated proteins such as fascin actin-bundling protein 1 (FSCN1), cofilin-1 (CFL1), and stathmin 1 (STMN1) was also observed, together with upregulation of the neurogenesis-related proteins TATA-box binding protein associated factor 1 (TAF1), septin 2 (SEPT2), non-phototropic hypocotyl 3 (NPH3), and cellular retinoic acid-binding protein (CRABP) (Chae et al., 2009). Cytoskeletal organization is a dynamic process that governs critical events essential for neurogenesis, such as cell division, migration, and differentiation (Compagnucci et al., 2016). Thus, changes in protein expression levels correspond to the shifting structural and functional demands of neural cells. A later study found 118 DEX proteins between hESCs and differentiated NSCs. Gene ontology analysis revealed that proteins associated with RNA processing and protein folding were more abundant in undifferentiated hESCs. In contrast, proteins related to redox homeostasis, energy metabolism, and proteolysis were enriched in differentiated NSCs. Notably, enzymes involved in glycolysis and amino acid synthesis showed increased abundance during differentiation, indicating metabolic shifts necessary for the transition to neural cell types (Fathi et al., 2014). These findings indicate that the transition from hESCs to NSCs shifts away from transcriptional and translational processes toward greater dependence on metabolic pathways to facilitate neural differentiation. However, a contrasting study reported a decrease in glycolysis-related proteins during the induction of neurons from hESCs, while confirming an increase in proteins related to oxidative phosphorylation. This study also highlighted the role of autophagy-related proteins in organelle remodeling during neurogenesis, suggesting active turnover of unnecessary organelles as part of the differentiation process (Ordureau et al., 2021; **Table 1**). These findings point to major changes of the proteomic profile during neural differentiation and the critical role of cytoskeletal reorganization and metabolic adaptations in this process.

**Table 1:** Protein expression patterns during embryonic brain development

Pluripotent cells	Differentiated cells	Days post-induction	Total proteins	Differential expression	Functions/proteins	Methods	References
Midbrain stem cell line (hNSC)	Neurons	0, 4, and 7	318	45	Cell cycle, transcription, nucleic acid transport and metabolism, protein synthesis, metabolism, processing and degradation, signal transduction, stress response, energy metabolism, carbohydrate metabolism and transport, cytoskeletal	MS	Hoffrogge et al., 2006
Neuroectodermal spheres (hNSC)	Neural progenitors	7	68		28 upregulated proteins: cytoskeletal organization, oxidoreduction-associated proteins, neurogenesis, neural development, and lipid binding activity	2DE > MS	Chae et al., 2009
hESC	Neurons	2 (neural induction) 9 (neuroectoderm) 30 (differentiated)		118	Enriched in hESCs and differentiated neurons: GTPase signal transduction and neurotransmitter transport Enriched in differentiated neurons: redox homeostasis and retinoic acid signaling Enriched in neuroectoderm and differentiated neurons: metabolic process, redox homeostasis, protein ubiquitination, and transport Enriched in induction: metabolism, redox homeostasis and transport Enriched in hESCs: RNA processing Enriched in hESCs and induction: adherent junctions, protein folding	2DE > MS	Fathi et al., 2014
hESC	Neurons	12			Downregulated: transcription factors associated with cell cycle regulation and associated to the maintenance of pluripotency. Upregulated: transcription, synaptic function, and neuronal morphology.	2DE > MS	Ordureau et al., 2021
hESC	Astrocytes Motor neurons	8 time points within 3 wk	1185		Embryoid bodies: SPTBN5, NARF, GAA, RPL11, NUCKS1, MRPL18, and EPB41L2 Embryonic stem cells: FABP5, DSP, PYGL, GARS, TRAP1, RPSA, SLC3A2, SLC7A5, SERPINB9, and LMCD1 Astrocytes: SUPT6H, FN1, HBA1, TNC, DIXDC1, PLS3, and DLST Early stages of motor neuron differentiation: HIST1H1E, KPNA4, HIST1H1C, CRABP2, LOC732081, HIST2H2AB, YWHAQ, TUBA1B, KRT8, UPF1, KRT18, FDPS, and HSPB1 Progressive increase in motor neurons: NEFM, DCLK1, SKP1, RBP1, COTL1, TMSB10, CKAP4, AKAP12, VIM, POSTN, RCN1, CFL1, CALR, and MAP1LC3B2 Progressive decrease in motor neurons: PTMA, APOE, SKIV2L2, and HMCN1, CNDP2, C10orf119, LDHA, PIK3CA, BAT2D1, CNPY2, ALPL, DNMT3B, FANCI, and AASS	MS	Chaerkady et al., 2009
hESC	NPC > GPC > OPC	25	3145		Myelination-related proteins: Upregulated in NPC: SNAP23, SBF1, CREB1, GNB2L1, LPCAT1, SOX2, CD44, SOD1, and TNIK Upregulated in GPC: SNAP23, SBF1, CREB1, LAMB1, CLU, TF, AXL, GNB2L1, and NPC2 Upregulated in OPC: SNAP23, SBF1, MARCKS, VIM, LGALS1, HSPB1, CRYAB, FABP4, APOE, TGFBI, THBS1, AHNAK, COL3A1, C14orf142, SCARB2, NPC2, ARSA, TF, NCAM1, IGFBP2, RELA, WNT5A, BMP1, COL6A1, and EPB41L3	MS	Chaerkady et al., 2011
hESCs	hNSCs phosphotyrosyl	7		12 up	RNA metabolism, processing, location establishment, splicing, transport and nucleic acid transport Nucleotide binding, DNA/RNA binding and protein binding	2DE > MS	Kim et al., 2011
hESCs	hNSCs phosphoproteome	6	13,000 (60,000 phopshorylation sites)		Enriched in hESC: structural and regulatory proteins Enriched in hNSC: chromatin modification, neurogenesis, axonal guidance, cell adhesion, androgen receptor signaling, and microtubules.	MS	Singec et al., 2016

2-DE: Two-dimensional gel electrophoresis; GPC: glial progenitor cells; hESC: human embryonic stem cells; hNSC: human neural stem cells; MS: mass spectrometry; NPC: neural progenitor cells; OPC: oligodendrocyte precursor cells.

The brain comprises a wide range of cell populations, each distinguished by unique morphology, physiological properties, and anatomical location (Ecker et al., 2017). Despite this complexity, few studies have focused on understanding the specific proteomic signatures of hESC differentiating into specific neural cell types. One study tracked protein expression across eight time points as hESC differentiated into motoneurons or astrocytes, revealing distinct patterns of abundance. Proteins such as S-100 and Tenascin-C were identified in astrocytes and, neurofilament-3, doublecortin (DCX), calmodulin-dependent protein kinase 1 (CAMK1) and Nestin in motoneurons (Chaerkady et al., 2009), highlighting the specialized roles of these proteins in providing structural support unique to each cell type. Furthermore, a study on the transition of neural progenitor cells into glial precursor cells and oligodendrocyte precursor cells identified a significant number of proteins potentially involved in oligodendrocyte maturation. From the neural progenitor cell stage, proteins associated with pluripotency were downregulated while proteins involved in myelination were upregulated. Additionally, nidogen-1 (NID1) a structural glycoprotein present in the basement membrane was suggested as a marker for glial precursor cells and several crystallin proteins as markers of oligodendrocyte precursor cells (Chaerkady et al., 2011; **Table 1**). These studies expand our understanding of lineage-specific differentiation and unveil promising markers that could serve for tracking neural development.

The importance of post-translational modifications (PTM) in modulating protein activity and molecular interactions is widely acknowledged in the omics fields. PTM refer to chemical modifications that proteins undergo after synthesis. Among these, protein phosphorylation, the addition of phosphate groups to protein residues, is one of the most extensively researched PTM and plays a critical role in regulating neuronal function (Johnson, 2009). The first phosphoproteomic assay on NSC revealed the upregulation of 12 proteins, 7 of which were involved in neuronal differentiation, emphasizing the abundance of cytoskeletal components and proteins involved in nucleic acid processing (Kim et al., 2011), which reinforces the critical roles of gene expression modulation and cytoskeletal reorganization during neural differentiation. A later study, provided further insights, identifying 13000 proteins with 60000 phosphorylation sites. Among these proteins, 76.4% were phosphorylated, including 75.1% of the 487 transcription factors detected. These phosphoproteins were linked to 415 biological pathways (Singec et al., 2016), confirming the broad functional scope of this PTM (**Table 1**).

Overall, these analyses highlight the temporal dynamics of the embryonic neural development, emphasizing the reduction of pluripotent markers during differentiation, the upregulation of cytoskeletal and cell cycle regulatory proteins, and the emergence of cell specific proteins in later stages of differentiation.

### Fetal development

The complexity and regional specificity of the fetal brain, combined with the ethical challenges associated with obtaining tissue samples, restrict the number of studies conducted in this gestational age. Despite these challenges, a study provided a deep understanding of regional development across gestational weeks 16 to 36 by examining protein levels in the ventricular zone, intermediate zone, subplate and frontal cerebral cortex using Formalin-Fixed Paraffin-Embedded tissue. This analysis identified a total of 3041 proteins, with 609 DEX between the PFC and different ventricular cellular layers, showing an enrichment of synaptic proteins in the PFC and DNA replication processes in the ventricular areas. This study showed that proteomic techniques can capture the dynamic, region-specific development of the brain, supporting the creation of molecular atlases that map the programs involved in brain development (Djuric et al., 2017; **Additional Table 1**).

**Additional Table 1:** Protein profiles across brain regions and developmental stages

		Fetal development					Postnatal development				
	Region	Reference	GW	Protein	Functions	Method	Reference	Age	Protein	Functions	Method
**Ventricular**	**VZ**	Djuric et al., 2017	16-36	2917	609 DEX ventricular - cortical Enrichment ventricular: DNA replication processes Enrichment cortex: of synaptic assembly and transmission 306 DEX NPC-Neurons Upregulated in NPC: neuronal function or maturation Downregulated in NPC: synaptic function and glutamate signaling Upregulated in neurons: neurite outgrowth, markers of neural differentiation Downregulated in neurons: endoplasmic reticulum proteins involved in translation initiation and elongation	MS					
	**IZ**			2957							
	**SP**			2927							
**Cortical**	**PFC**	Djuric et al., 2017	16-36	2989		MS					
		Zhao et al., 2022	9, 11, 13	2650	9 DEX GW9-GW11: neuronal development, differentiation and neurogenesis, neuronal activity. 33 DEX GW11-GW13: hydrogen peroxide catabolic processes, transport and localization, neurogenesis, neuronal migration, axonal growth.	MS					
		Wang et al., 2023	18-41	1765 PSD	-Translation related-pathways -Axon guidance -Rho GTPase pathways (GW28-41)	MS	Wang et al., 2023	< 18 yr	1765 PSD	-Synaptic transmission -neurexin-, neuroligin-associated pathways -Rho GTPase pathways (0-1 yr)	MS
	**DLPFC**						Carlyle et al., 2017	Birth-42 yr	7778	SERPINF1, NGEF, CDH8, PRPS2, RGS6, RFTN1, C2CD4C, TPBG, DGKB, KIAA1107, PPP1R14C, CYP46A1, ITPR3, RASGRF2, PDE4D, H1FX, PDE4D, FAM131B, S100A1, CRTC1, and TYRO3	MS
							Breen et al., 2018	39 d - 49.5 yr	911	60 PSD proteins overexpressed in early development. 99 proteins involved in cellular respiration and GTPase binding overexpressed in adulthood. 39-89 d period larger level of variability. Functions enriched CNS development, neurogenesis and gliogenesis.	MS
	**V1C**	Wang et al., 2023	18-34	1766	-Translation related-pathways -Rho GTPase pathways (GW28-41)	MS	Wang et al., 2023	< 18 yr	1766	-Rho GTPase pathways (0-1 yr) -Synaptic transmission	MS
							Carlyle et al., 2017	Birth - 42 yr	8146	MTFR2, CRTAC1, MYO1B, KIAA1107, ANK1, RGS12, RCC2, KHDRBS3, NTNG2, OGN, SNRPA1, DCPS, TCEAL3, GPCPD1, GRIN2A, ARID2, PVALB, TYRO3, SCN1A, and ATXN2	MS
**Cerebellar**	**CBC**					2-DE	Carlyle et al., 2017	Birth - 42 yr	8208	HIST1H1B, H2AFY2, CBLN1, FAT2, SLC1A6, GRID2, CA8, H1FX, ATP2A3, HOMER3, PCP2, SCGN, EPB41, GRM4, TRIM72, CEP76, HIST1H1C, PVALB, ARHGEF33, and GRID2IP	MS
**Subcortical**	**STR**						Carlyle et al., 2017	Birth - 42 yr	7930	Dopaminergic signaling, drug addiction PDE1B, GNAL, PDE10A, NGEF, DGKB, ENTPD3, PTPN5, ADCY5, PENK, TH, PPP1R1B, ACHE;CES4A, SLC6A3, LAMP5, ANKRD63, RGS14, TPBG, ARPP21, TDP52L1, and SCN4B	MS
	**MDT**								7754	BTBD17, AKAP7, AKAP2, HOMER2, LRRTM1, CDH6, AKR1C2, ADCY8, TRPM3, B3GALTL, SLC6A9, SIAE, GMPR, PVALB, CCDC136, CLMN, SYT6, SLC6A11, PLCB4, and CPNE9	
	**HIP**								7996	SMPD3, PPM1E, NPTXR, LPPR2, DOCK4, SCN3B, CACNG8,NRP1, PTPRK, SPHKAP, RGS14, IGF2BP2, TOMM34, SCGN, WIPF3, ASNS, ARHGEF6, KCTD4, PDYN, and CAPS	
	**AMY**								7804	NPTXR, PLXNC1, BABRA2, RAB3B, PCDH19, SYT17, NTRK3, CACNG8, MFSD4, SFN, GPC4, LRFN1, KCTD4, KCNG3;KCNA4, GPRASP1, GSTM5, FXYD7, PDYN, CAPS, and SCOC	
**Vascular**	**BBB**						Zhou et al., 2024	34 GW> 60 yr	6223	Protein transport, cell adhesion, cell junction, endosomal transport, cell-matrix adhesion, extracellular matrix organization, neurotransmitter transport, cytosolic transport, cytoskeleton-dependent intracellular transport	MS

2-DE: 2-Dimensional gel electrophoresis; AMY: amygdala; BBB: blood-brain barrier; CBC: cerebellum; CNS: central nervous system; DEX: differential expression; DLPFC: dorsolateral prefrontal cortex; GW: gestational week; HIP: hippocampus; IZ: intermediate zone; MDT: medio-dorsal thalamic nucleus; MS: mass spectrometry; NPC: neural progenitor cell; PFC: prefrontal cortex; PSD: post-synaptic density; SP: subplate; STR: striatum; V1C: primary visual cortex; VZ: ventricular zone.

Functional development has also been characterized by Want et al., who created a proteomic map profiling the temporal dynamics of more than 1000 proteins in post-synaptic densities from the prefrontal cortex and primary visual cortex. Major differences were observed in excitatory and inhibitory post-synaptic densities, which may contribute to different post-synaptic responses in different types of neurotransmissions. Notably, translation-related pathways and axon guidance were overrepresented in the prenatal period, while Rho GTPase pathways, involved in cytoskeletal remodeling, were overexpressed perinatally (Wang et al., 2023; **Additional Table 1**).

Furthermore, brain asymmetry is also reflected in the proteomic profile of each brain hemisphere, as shown by Zhao et al. (2022) between gestational weeks 9 to 13. Protein expression changes were observed in the bilateral frontal lobes, with more pronounced asymmetry during first stages of gestation. These findings suggest that lateralization occurs during early brain development and emphasize the need of conducting omic studies separately on each hemisphere before lateralization occurs (Zhao et al., 2022; **Additional Table 1**).

Despite tissue heterogeneity, a substantial proportion of proteins detected in the mentioned analyses were related to protein synthesis, cytoskeletal dynamics and cell cycle regulatory functions. This aligns with findings from embryonic brain development, underscoring significant changes and continuous maturation throughout all stages of gestation.

### Postnatal development

In early postnatal development, differentiated CNS structures exhibit different dynamics of gene expression and protein abundance to meet the biological demands of each region. As development progresses, protein profiles continue to reflect ongoing structural and functional changes. The investigation conducted by Wang et al. (2023) on proteins from post-synaptic densities also explored postnatal development through to adulthood. As mentioned, they found overrepresentation of Rho GTPase pathways shortly after birth and subsequent increase in synaptic transmission and neurexin-, neuroligin-associated pathways, involved in synapse formation, that persisted until adulthood. Their proteomic data also revealed the sequential activation of protein categories defining three major stages of synapse formation, corresponding to fetal, perinatal and postnatal development (Wang et al., 2023). A study conducted by Carlyle et al. (2017) on seven brain regions (cerebellum, striatum, medio-dorsal thalamic nucleus, amygdala, hippocampus, primary visual cortex and dorsolateral prefrontal cortex) found 1804 DEX proteins between one or more regions and, 123 DEX proteins across developmental periods ranging from infancy to adulthood. Functional clustering of proteins revealed region-specific expression patterns emphasizing the enrichment of nuclear proteins in the cerebellum, a neuron-dense region, while cortical regions showed enrichment for Gene Ontology terms related to receptor activity and plasma membrane localization. In the striatum, protein clusters related to dopaminergic signaling and addiction pathways were prominent. This work enabled the creation of a peptide library of the seven brain regions analyzed. The number of DEX observed varied more between brain regions than across developmental periods, probably reflecting that major developmental changes occur in earlier stages of brain development. In support of this hypothesis, some DEX were unique to infancy and were enriched for clusters comprising proteins related to neuropsychiatric and neurological disorders. However, the small sample size per age group (16 individuals, 1 to 42 years) may limit the ability to detect differences across developmental stages (Carlyle et al., 2017; **Additional Table 1**).

Indeed, infancy is a critical period for cortical brain development and, alterations in the temporal dynamics of protein levels during this period have been associated with neurodevelopmental disorders. One study looked at protein abundance in the dorsolateral prefrontal cortex from postnatal development to adulthood, finding a time-dependent upregulation of proteins related to gliogenesis, myelination, oligodendrocyte specificity, NADH (reduced nicotinamide adenine dinucleotide) metabolism and gluconeogenesis, while proteins related to axonogensis, cytoskeletal dynamics and neuronal cell types were downregulated. This pattern suggests an early developmental phase dominated by neuronal growth and network formation, followed by a later phase characterized by circuit stabilization and the metabolic support required to maintain these networks. A key finding of this study was the identification of DEX groups during brain development that overlapped with genetic risk loci for neurodevelopmental disorders, emphasizing the relationship between the genetic makeup and protein expression patterns underlying neurodevelopmental pathology (Breen et al., 2018; **Additional Table 1**).

Besides, a proteomic analysis of the brain microvasculature identified a total of 56 solute carriers (SLC) involved in nutrient transport, pointing to developmental shifts in energy demands. Early stages of brain development showed a greater reliance on amino acid transport, consistent with the higher CSF/plasma amino acid ratio observed during childhood (< 3 years) (Scholl-Burgi et al., 2008). Notably, several transporters, SLC5A6, SLC19A3, and SLC52A3, which had not been previously reported in brain proteomic analyses were detected. Additionally, DEX of structural proteins across development also shed light on the maturation of the brain vascular system. Well-stablished structural proteins such as zonula occludens-1 (ZO-1), claudin-5 (CLDN5), occludin (OCLN), and vascular endothelial (VE)-Cadherin maintained stable expression across time points, whereas claudin-11 (CLDN11) and integrin beta-1 (ITGB1), both crucial for the maintenance of blood-brain barrier integrity, correlated positively with age (Zhou et al., 2024; **Additional Table 1**).

The progressively dynamic patterns of protein expression across the CNS structures reflect the complex and evolving physiological requirements that drive the compartmentalization of the brain and the functional specialization of its regions.

### Organoids

As mentioned above, the limited availability of human cerebral tissue and ethical constraints on its obtention pose significant challenges to characterizing brain proteomic profiles across developmental stages and under specific pathological conditions. Brain organoids are biological structures derived from hPSCs that replicate the 3D organization of brain tissue and the cellular diversity, spatial organization, and functional interactions of the developing brain (Kim and Chang, 2023).

In a comparative study, Nascimento et al. (2019) demonstrated that human cerebral organoids closely resemble fetal brain tissue. Both exhibited developmental features such as RNA regulation, adenosine triphosphate metabolism, and cytoskeletal organization, processes essential for neuronal morphogenesis and neurite outgrowth. Brain organoids also displayed cell-specific protein profiles enriched in proteins related to neurogenesis, axon guidance, and synaptogenesis, which reflect cortical development and proteins involved in metabolism and cell signaling that mirrored glial protein landscapes (Nascimento et al., 2019). Extended analysis showed that cerebral organoids recapitulate key aspects of the fetal brain proteome at gestational weeks 16 to 20 with approximately 40% proteomic overlap and shared proteins across all cortical layers, underscoring the developmental relevance of these models (Djuric et al., 2017; Nascimento et al., 2019). Another study further emphasized the cellular heterogeneity within brain organoids, finding cell-specific proteomic profiles that overlapped those found in fetal brain tissue at mid-gestation. At day 40 of culture, the organoids displayed SRY-box transcription factor 2 (SOX2)-positive and DCX-positive cell subpopulations, which showed different protein abundances with 322 and 158 DEX, respectively. Interestingly, in the SOX2 subpopulation the protein landscape remained stable over time, consistently enriched for proteins involved in DNA-replication and cell cycle and correlating with ganglionic eminence regions in the human fetal brain. In contrast, the DCX compartment exhibited temporal changes, primarily affecting signal transduction and regulatory pathways with the proteomic program overlapping with ganglionic regions as well as the dorsal subventricular zone, cortex and striatum (Melliou et al., 2022). SOX2 as a marker of neural progenitor cells and DCX as a marker of newborn neurons have been well documented during early human brain development (Eze et al., 2021). Yentur et al. (2025) further analyzed proteomic changes over time in human dorsal forebrain organoids. At day 20, organoids displayed increased levels of proteins linked to DNA replication and cell division, indicative of proliferation. By days 35 and 50, proteins associated with differentiation and synapse formation emerged, reflecting a transition to functional connectivity and network assembly (Yentur et al., 2025).

Brain organoids also provide a valuable model for studying PTMs throughout development. As shown in proteomic analyses of hESCs and NSC, PTMs play a crucial role in regulating embryonic human brain development (Kim et al., 2011; Singec et al., 2016). Recently, a study characterized protein abundance and PTMs in brain organoids at 28 developmental time points, identifying 9300 proteins, along with key neurodevelopmental PTMs such as phosphorylation, acetylation and cysteine modifications, which seemed to be critical for protein folding and cellular signaling. Around 40% of these proteins showed temporal-dependent expression patterns similar to fetal development, with increased abundance of proteins associated with cell proliferation, RNA splicing, and chromatin remodeling related to progenitor cells, and proteins linked to metabolic pathways and synaptic function during neuronal maturation. These groups of proteins also exhibited dynamic PTMs, including the phosphorylation of proteins involved in axon guidance, which temporarily coincided with the increased abundance of synaptic proteins (B. Elmkvist, 2024). Together, these findings indicate that brain organoids not only capture region-specific proteomic profiles but also recapitulate the temporal maturation of the proteome observed in human brain tissue, highlighting their value as models for studying brain development and regional specialization.

## Identification of Pathological Signatures of Fetal Growth Restriction through Proteomic Profiling of Body Fluids and Fetal Compartments

**Figure 1:**
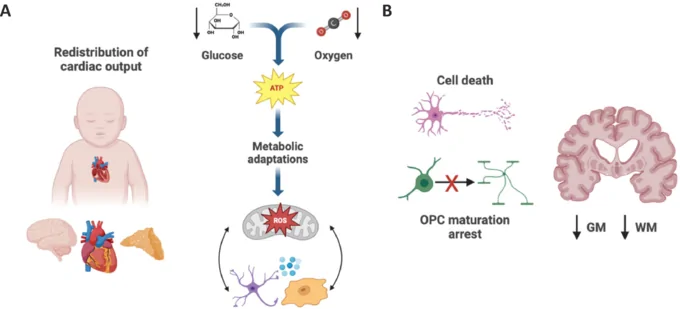
Pathophysiology of fetal growth restriction. (A) In response to fetal growth restriction, the organism adapts by redistributing cardiac output into central organs, such as the brain, heart and adrenal glands. Long-term deprivation of oxygen and nutrients triggers a series of metabolic adaptations, with the subsequent production of reactive oxygen species and the activation of an inflammatory response. (B) These alterations lead to neural cell death and hinder the maturation of oligodendrocyte precursor cells (OPC) into myelinating oligodendrocytes, resulting in macrostructural changes, characterized by decreased volumes of grey matter (GM) and white matter (WM). Created with BioRender.com.

Chronic deprivation of oxygen and nutrients causes a series of physiological adaptations aimed at preserving fetal survival. The first compensatory mechanisms entails a hemodynamic response characterized by increased vasodilatation in critical organs, such as the heart, brain and adrenal glands, while vasoconstriction predominates in less vital peripheral tissues (Ramirez Zegarra et al., 2022; **Figure 1A**). This results in a ‘brain sparing’ effect, where cerebral vascular resistance is reduced to enhance perfusion, directing blood flow primarily to frontocortical regions irrigated by the anterior cerebral artery. As pathology progresses, perfusion shifts towards deep grey matter structures supplied by the middle cerebral artery (Steller et al., 2022). Thus, structures responsible for higher functions are prioritized before transitioning to those essential for survival.

The prolongation of this event leads to profound metabolic changes. When glucose availability becomes insufficient, the organism increasingly relies on alternative substrates, including fatty acids and amino acids, which are mobilized for gluconeogenesis (Melkonian et al., 2025). As this condition worsens, there is a switch to anaerobic metabolism, with increased lactate levels and the subsequent development of metabolic acidosis, which further impairs cellular homeostasis (Cetin et al., 2020). These metabolic alterations lead to the production of reactive oxygen species, weakening the antioxidant defenses, which in turn leads to oxidative damage of proteins, lipids and nucleic acids (Rains et al., 2021). Persistent oxidative stress not only compromises mitochondrial integrity, but also accelerates cell death (Jakubczyk et al., 2020). These molecular events trigger a robust inflammatory response. Activated microglia and astrocytes infiltrate affected regions, releasing a complex mixture of cytokines and chemokines. The imbalance between pro-inflammatory mediators and anti-inflammatory signals amplifies tissue injury and interferes with normal neurodevelopment (Wan et al., 2021; **Figure 1A**).

This chronic hypoxic injury may cause reduced grey matter volumes due to neuronal cell loss and maturation arrest of oligodendrocyte precursor cells (Reid et al., 2012; Dudink et al., 2022). This results in loss of microstructural integrity, diffuse white matter injury, and alterations in major fiber tracts (Mayrink et al., 2024). The MRI study has also shown reduced fractional anisotropy in corticospinal and association pathways (Eixarch et al., 2016), suggesting impaired axonal development and connectivity (**Figure 1B**). At the macrostructural level, children exposed to a hypoxic intrauterine environment frequently exhibit disrupted patterns of brain maturation, with altered cortical gyrification and sulcation patterns (Dubois et al., 2008), reflecting disturbed neuronal migration and cortical organization during gestation. These macrostructural alterations have been strongly correlated with adverse neurological outcomes (Dubois et al., 2008; Mayrink et al., 2024; **Figure 1B**).

Proteomic profiling of umbilical cord blood, amniotic fluid and placenta has been used to characterize growth restricted pregnancies. In this regard, a study on umbilical cord serum and amniotic fluid of full-term newborns identified 14 and 11 proteins with DEX, respectively. These proteins were primarily involved in coagulation, blood pressure, immune responses and ion homeostasis (Cecconi et al., 2011), mechanisms that have been associated with the pathogenesis of FGR (Chinni et al., 2010; Mullins et al., 2012). This study validated five proteins in umbilical cord serum complement 3, angiotensinogen, transferrin, kininogen, clusterin, and showed that even if detectable in maternal blood, they lack biomarker potential. Complement 3 and angiotensinogen levels can be increased in response to other causes of inflammation or hypertensive disorders, whereas transferrin levels are strongly influenced by maternal circulating ion levels. kininogen and clusterin are less abundant and exist in multiple isoforms, challenging their detection and reproducibility. Further proteomic investigation emphasized the role of apolipoproteins and lipid metabolism in FGR infants born preterm. Elevated triglyceride levels, a common feature of FGR pregnancies, were linked to alterations in the number of sialic acid residues bound to apolipoprotein C. Specifically, apolipoprotein C-III₀, the least sialylated isoform and a known marker of hypertriglyceridemia, was identified as the predominant form in FGR cord serum (Maeda et al., 1994; Wolter et al., 2012). However, the maternal metabolic status could influence the sialysation of apolipoprotein C, limiting its biomarker potential. Interestingly, a recent study in cord plasma uncovered sexual dimorphisms in the proteomic signature of FGR infants born term, identifying 36 proteins with DEX between males and females, and 29 proteins showing an interaction between growth and sex. They highlighted DEX of peroxisomal proteins between controls and FGR. However, this pathway seemed to be affected in females but unchanged in males. Other metabolic proteins associated with obesity, diabetes and glucose intolerance were more prominently altered in males. Due to the increased representation of peroxisomal proteins, the results of the proteomic study were validated measuring catalase activity. Even if the activity was increased in FGR, this increase was significantly prominent in females, generating a sex difference that hinders the use of this assay as a biomarker for FGR (Akinyemi et al., 2024; **Table 2**).

**Table 2:** Proteins in human samples of fetal growth restriction

Sample	Method	Time	Total	Upregulated	Functions/proteins	Downregulated	Functions/proteins	Biomarker validation	References
Umbilical cord	2-DE	Birth		5 spots	Lipid transport, coagulation, oxygen transport, cytoskeleton organization, iron transport	13 spots	Complement pathway, vasoconstriction, inflammatory response, immune response, cytoskeleton organization	C3, TF, KNG1, CLU, AGT	Cecconi et al., 2011
Blood	MS	Birth	852	62	Peroxisomal transport, organization, and localization, electron transport chain, nucleotide metabolism, catabolic processes Females: Peroxisomal transport, organization, and localization, oxygen transport, monosaccharide and glycosul metabolism Males: -	15	Platelet degranulation and aggregation, cell adhesion, regulation of response to stimuli Females: Platelet degranulation and aggregation, cell-matrix adhesion, complement activation and ERK1 and ERK2 cascades. Males: peptidyl-tyrosine phosphorylation, LDL mediated signaling	Catalase activity	Akinyemi et al., 2024
	MS	Birth	+ 60 ion signals	Lipid transport and metabolism				Apolipoprotein C-III	Wolter et al., 2012
Umbilical cord Wharton’s Jelly tissue	MS	Birth		10	GSR, ACAN, SDH, DCXR, ADAM10, ADGRE5, GNB4, GGT5, FBLIM1, STAT6	23	MXRA5, PZP, CILP1, P3H1, COL11A1, COL5A3, THBS3, FAP, RPS14, SRPX, SSR, MSLN, CDH11, RCN2, IGHG4, COL16A1, AFP, PLOD1, OLFML3, ISOC1, SPON1, MATN2, COL1A1	Not performed	Conrad et al., 2022
Amniotic fluid	2-DE	Birth		7 spots	Carbone metabolism, blood coagulation, immune response, transport	4 spots	Protein binding, ion transport, inflammatory response, transport	Not performed	Cecconi et al., 2011
Placenta	MS	Birth	356	Proteins in control placenta: HIST2H2BF, SERPINA1, ANXA2, ANXA5, ACTC1, ACTA2, ATP5F1B, CSH1, ANXA1, ACTN4, VCL, COL14A1, MYH9, FLNA, TLN1, AHNAK Proteins in FGR placenta: HIST2H2BF, SERPINA1, ANXA2, ANXA5, ACTC1, ACTA2, ATP5F1B, CSH1, ANXA1, ACTN4, VCL, COL14A1, MYH9, FLNA, TLN1, AHNAK, APOA1, PRDX2, YWHAZ, TPI1, LDHB, CAT, HPX, CALR, P4HB, MSN, PKM, TKT, CP, TUBB2B				Not performed	Geca et al., 2022
Mother blood	MS	Prior C-sec	166	14	Complement and coagulation cascades, lipid metabolism and transport	17	Endopeptidase activity, secreted plasma proteins	SERPINA3 marker of Preclampsia+FGR	Auer et al., 2010
	MS	2 h post-delivery	688	9	APOE, GP1BA, APOC3, THAP4, APOC2, PSG9, TAGLN2, PSG11, LGALS3BP	16	FGA, P04220, OLFML2B, ATP5L, DEFB103A, FABP5, CTBS, EGFR, PRG4, P01815, PSG2, PLTP, LGALS7, LTF, ADIPOQ, LYVE1	NOTCH1, mediator of FGR severity. Validated in a subsequent study (Ye et al., 2025).	Paules et al., 2020
Infant blood	2-DE	During mon 1	661	Differential expression in FGR: ALBU, GSN, APOE, KRT10, SLC41A2, ANAPC2, TF, FCN2, APOA1, UGP2, SERPIN1, DNAH6, APOH, VTN, RAB35, APOH, PRDX2, FGB Present only FGR: MBOAT7 Present only AGA: SUMO3, APOL1, DEFB108P1, IGLC6, IGLC2, KRT9, CCDC51				MBOAT7, purposed as marker of an adaptative response. Validation not performed.	Ruis-Gonzalez et al., 2015

2-DE: Two-dimensional gel electrophoresis; AGA: appropriate for gestational age; FGR: fetal growth restriction; MS: mass spectrometry; Time: time of sample collection; Total: total of proteins detected.

Moreover, a histological analysis of the umbilical cord revealed a larger cord diameter in controls compared to FGRs born at a mean gestational age of 35 weeks, driven by a reduction in Wharton’s Jelly area. In cord tissue, 11 DEX proteins involved in oxidative stress, immune response and cell adhesion were upregulated, while 23 related to the extracellular matrix, RNA processing, immune response, and cell function and death were downregulated (Conrad et al., 2022). In the placenta of FGR pregnancies, proteins related to similar pathways, including oxidative stress, apoptosis, cell differentiation and proliferation and cellular functions, showed differences in abundance. In this case, evidence of disrupted lipid metabolism was detected (Geca et al., 2022; **Table 2**). Umbilical cord and placental abnormalities are among the most common causes of FGR, and the molecular alterations identified in these proteomic analyses point to compromised tissue integrity and cellular homeostasis in FGR fetal structures.

Proteomic analyses on maternal and infant blood complicated by FGR provided deeper insights into the mechanisms contributing to this obstetric complication. An early study applying Isobaric Tags for Relative and Absolute Quantitation technology on maternal blood from FGR and preeclampsia pregnancies allowed the identification of proteins previously undetected in these conditions. A large proportion of these belonged to the Serine Protease Inhibitor (SERPINA) family, which are proteins associated with severe preeclampsia (Starodubtseva et al., 2020). In total, 166 proteins were detected, out of which 31 were modified in FGR maternal blood and 28 in FGR with preeclampsia. Notably, the protein expression profile was altered when both conditions co-occurred. Proteins involved in complement and coagulation cascades appeared to be characteristic of FGR and more strongly linked to placental injury than to changes in maternal blood pressure. In contrast, acute phase proteins, involved in inflammation, were upregulated in preeclampsia but downregulated when both pathologies were present, pointing to a feto-placental mechanism to regulate the inflammatory response. Of these proteins, SERPINA3 protein levels were validated by Western blot and, found to be increased in FGR pregnancies with preeclampsia (Auer et al., 2010). SERPINA3 is not a useful biomarker of FGR, but a previous study has also found its utility as a biomarker of preeclampsia in placental tissue (Chelbi et al., 2007). Consistent with previous findings, analysis of maternal blood collected at birth from pregnancies with late onset-FGR revealed DEX of 25 proteins primarily related to lipid metabolism. Notably, neurogenic locus notch homolog protein 1 was identified in maternal blood as a potential modulator of the FGR phenotype and proposed as a novel biomarker (Paules et al., 2020). Despite this study not conduction subsequent analysis for the validation of the proteomic data, a very recent study has validated Noth signaling pathway as a regulator of the FGR response by mediating the immune response (Ye et al., 2025). In blood of infants, differences in protein abundance were observed across term, moderate preterm, and very preterm FGR cases, as well as compared to infants born appropriate for gestational age. A total of 33 proteins involved in protein transport, coagulation, immune response and cytoskeletal organization displayed differential abundance. That indicates that growth restriction exerts systemic effects that vary with gestational age. These widespread alterations can be detected in the blood proteome and, may serve as potential biomarkers to monitor FGR. In this regard, some of these proteins were undetectable in FGR cases at any gestational ages, whereas membrane-bound O-acyltransferase domain-containing 7 (MBOAT7) upregulation, only detected in FGR, was proposed as a biomarker of an adaptive response to FGR (Ruis-Gonzalez et al., 2015). However, further validation of the proteomic data would be required to further assess the biomarker value of this protein (**Table 2**).

These findings emphasize convergent mechanisms across tissues and body fluids derived from FGR samples, underscoring dysregulation of oxidative pathways, immune responses, coagulation, lipid metabolism, and cellular processes, aligning with the known pathophysiology of this condition. Additionally, these analyses identified potential biomarkers with diagnostic and prognostic potential. However, in this discussion, we just consider proteins subsequently validated, as mass spectrometry detects peptides derived from protein digestion that may lead to false-positive identifications or overestimation of biological significance. Additionally, most of potential biomarkers detected present pitfalls, as they could also be regulated by other medical conditions not related to FGR, leading to diagnostic errors. Furthermore, additional technical issues should be considered, such as the concentration levels of the proposed proteins and the availability of sensitive assays to detect them.

## Neuroproteomic Profiles in Animal Models of Fetal Growth Restriction

Rodents are the most used animal models in biomedical research due to their low cost, high reproduction rate and, genetic homology with humans. However, inherent physiological differences limit the translatability of findings (Dominguez-Oliva et al., 2023). While modeling disease in large mammals entails methodological challenges and higher costs, their anatomical and physiological similarities make them valuable candidates for modeling human diseases (Hou et al., 2022). Domestic pigs are among the most used large mammals. Their gyrencephalic brains share comparable cortical organization, similar white matter composition (**~**60%), and similar regional distribution of neurotransmitter systems (Lunney et al., 2021; Netzley and Pelled, 2023).

**Figure 2:**
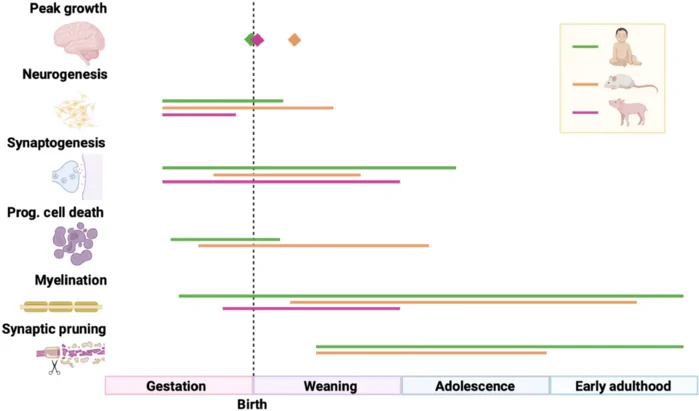
Timeline of brain development across species. Brain development encompasses multiple molecular processes, which include neurogenesis, synaptogenesis, programmed cell death, myelination and synaptic pruning. The onset and progression of these processes vary across species, determining the maturity of the central nervous system. This figure illustrates peak brain growth and the developmental stages at which key molecular events occur in humans (green), rodents (orange) and pigs (pink). Created with BioRender.com.

Neurodevelopment in humans, rodents and pigs progresses through comparable stages, albeit with notable differences in timing and duration. In humans and pigs, peak brain growth occurs perinatally, whereas in rodents, it peaks postnatally (Namburete et al., 2023). Neurogenesis begins in the embryonic period in all three species, ending postnatally in humans and rodents, but concluding mid-gestation in pigs (Glatzle et al., 2017; Zeiss, 2021). Synaptogenesis and programmed cell death occur during the gestational stage in humans and rodents, with synaptogenesis continuing until adolescence in both species, while programmed cell death continues through the early postnatal period in humans, but it concludes during adolescence in rodents (Zeiss, 2021). Similar to humans, the onset of synapse formation occurs around mid-gestation in pigs, although it ends earlier in the weaning period (Sobierajski et al., 2025). In humans and pigs, the onset of myelination occurs in the fetal period, but postnatally in rodents, continuing into early adulthood in humans and rodents, though concluding earlier in pigs (Sobierajski et al., 2023). Finally, synaptic pruning begins at weaning period in both humans and rodents, extending until adolescence in rodents and early adulthood in humans (Zeiss, 2021; **Figure 2**).

Understanding brain development across species is essential for translational research. Comparing species-specific differences facilitates the selection of appropriate animal models, determines the optimal timeframe to conduct analyses and assesses the extent to which findings can be translated to humans (**Table 3**).

**Table 3:** Comparative overview of species used in neuroproteomic studies of fetal growth restriction

	Rats	Pigs	Humans
Gestation	Short; 21–23 days	Long; 114 days	Long; 280 days
Placenta	Hemochorial	Epitheliochorial	Hemomonochorial
Neurodevelopment	Rapid brain growth; mainly postnatal	Gradual; prenatal and postnatal	Gradual; prenatal and postnatal
Brain morphology	Small size, lissencephalic cortex, simple regional organization	Large, gyrencephalic cortex, complex regional organization, high white-to-grey matter ratio	Large, gyrencephalic cortex, complex regional organization, high white-to-grey matter ratio
FGR induction	Experimentally induced; maternal undernutrition, placental insufficiency	Naturally occurring or experimentally induced, maternal undernutrition, placental insufficiency.	Naturally occurring; multiple ethnologies
Relevance for proteomic studies	Ideal for fundamental mechanistic insights	Suitable for temporal and tissue-specific studies	Direct relevance for human disease and biomarker validation
Considerations	Cost-effective, logistically simple, allows longitudinal studies, large sample sizes	More expensive, logistic challenges, limited time-points, small sample sizes	Ethical constraints, limited access to brain tissue
Translatability	Limited	High	Full

In FGR research, multiple animal models have been employed to characterize pathology and assess potential interventions, but proteomic analyses have primarily relied on rats and pigs. Rats provide practical advantages such as short gestation and accelerated neurodevelopment, which allow studies to be conducted in less time and at lower cost, enabling longitudinal studies and larger sample sizes. However, the inherent neurodevelopmental and anatomical differences limit translational relevance. FGR is typically experimentally induced, which can introduce confounding effects. Thus, while rats are well-suited for understanding the fundamental mechanisms affected by growth restriction, but are less suitable for direct clinical translation (Swanson and David, 2015; Netzley and Pelled, 2023). Pigs provide greater translational value due to their longer gestation and epitheliocorial placental that more closely resembles humans. FGR in pigs is often induced experimentally, but it can also occur naturally, reducing potential confounding effects. Additionally, similarities in organ development and brain morphology also allow for detailed, tissue-specific analyses. However, breeding and maintaining large mammals is logistically challenging and costly, limiting sample size and study duration (Netzley and Pelled, 2023).

### Rodents

In rodents, the analyses investigating the brain proteome of FGR offspring have mainly employed rats, where maternal nutritional restriction was used to induce the pathology. These analyses identified distinct biological functions contributing to brain abnormalities. One study found DEX of 25 proteins, primarily involved in metabolic processes and, enriched in pathways related to neurological and musculoskeletal disorders, cellular interactions and nervous system development (Liu et al., 2019), suggesting that FGR may impact key developmental and metabolic pathways, potentially contributing to long-term neurological and musculoskeletal impairments. In the same line, another study subjected female rats to calorie restriction during pregnancy. Proteomic analysis of brain tissue revealed the presence of proteins related to metabolic processes, oxidative status, and structural and enzymatic proteins. Notably, these proteins showed DEX between FGR pups and non-FGR siblings and controls, but not between non-FGRs and controls, indicating that reduced calorie intake alone does not necessarily result in FGR. This finding highlights the need to investigate the specific mechanisms that determine the development of FGR in offspring of undernourished mothers (Aravidou et al., 2016). More recent research provided deeper insights, revealing DEX proteins between all three groups (766 DEX FGR *versus* con / 648 in non-FGR *versus* con / 391 FGR *versus* non FGR), indicating that non-FGR siblings may also be at risk of developing neuropathological outcomes. These proteins were primarily involved in immune signaling (nuclear factor-kappa beta pathway), apoptosis, and angiogenesis (Potiris et al., 2021), pointing to shared mechanisms of inflammation, cell death, and vascular remodeling that may underlie long-term neurodevelopmental vulnerability. Despite the valuable insights obtained from these analyses, it remains unclear which brain regions contribute to the pathology, as they often refer to the analysis of brain tissue without precise regional specification. Supporting these results, proteome analysis of microglial cortical cells showed significant upregulation of nuclear factor-kappa beta, interleukins 1 and 17 pathways, and apoptosis at post-natal day (PND) 1 and PND4, whereas oxidative stress responding proteins were only upregulated at PND4 (Zinni et al., 2021). Alterations in the proteomic profile of the brain-resident immune cells further confirm that an abnormal inflammatory response is broadly involved in the neuropathology of FGR (**Table 4**). Overall, these findings underscore the role of metabolic processes, inflammation and proteins involved in neurodevelopment in the progression of the neuropathology caused by FGR. However, the oxidative stress response seems to be involved in later stages, likely due to a decline in antioxidant defenses.

**Table 4:** Proteomic profiles of brain regions in animal models of fetal growth restriction

Region	Species	Pathology induction	Sampling time	Method	Differentially expressed proteins	Functions/proteins	References
CTX	Rat	Low-protein diet	6 h post-partum	2-DE/MS	31	Protein binding, ion binding, hydrolase activity, nucleotide binding, lipid binding, enzyme regulator, structural molecule. Metabolic processes, biological regulation, multicellular organismal processes, response to stimulus, developmental process, cell communication, localization and proliferation, death.	Liu et al., 2019
	Rat	Nutritional restriction	post- partum	2-DE	13	Upregulated: signal transduction, cell cycle, Ca2+ signaling, protein folding, enzymatic activities, structural proteins Downregulated: axon guidance, protein folding, redox regulation	Aravidou et al., 2016
	Rat	Nutritional restriction	Post-partum	MS	FGR *vs*. control: 766 non-FGR *vs*. control: 648 FGR *vs*. non-FGR: 391	-Extracellular matrix, cell adhesion, protein transport, learning and memory, metabolism nuclear factor-kappaB signaling, apoptosis, angiogenesis, inflammation (FGR)-Cell migration and spreading (non-FGR)	Potiris et al., 2021
	Rat	Low-protein diet	PND1 PND4	MS	Control PND1 *vs*. PND4: 928 FGR PND1 *vs*. PND4: 20 FGR PND4 *vs*. control PND1: 243 FGR PND1 *vs*. control PND4: 429	PND1/PND4: nuclear factor-kappaB signaling, interleukin 1 pathway, hypoxia response, apoptosis, soluble exogenous antigens endosomes, non-canonical nuclear factor-kappaB pathway PND1: interleukin 17 pathway PND4: antigen presentation, lysosome vesicle biogenesis, oxidative stress	Zinni et al., 2021
HYP	Rat	Restricted access to food (50%)	4 mon post-partum	MS/2-DE	57	Downregulated: SUGT1, RAB5A, CD200, SYN1, COX6C2, ACADL, MAOB, HK2, COX7A2, RPS3A, SNAP25, AP2S1, PRKCB, MAP1LC3B, ALDH9A1, SEPT9, CTBP1, AVP, RAN, TXN, PPIA, NSFL1C, COX5B, SCG2, PCSK1N, MIF, CALB2, UBE2N, MYL6, ATP5B, ALDOA, ERP29, PGRMC1, DLD, UCHL1 Upregulated: ESD, STK39, ATP2B1, GSS, KCNAB2, GRB2, GSR, ROGDI, EIF3G, CIAPIN1, NUDT2, GPM6B, NDRG4, GSTO1, AKR1A1, CNP, CNRIP1, ARL8B, OGDH, ATP6V1B2, ATP5A1	Pedroso et al., 2017
	Rat	Protein restricted diet	PND12, PND16	2-DE, MS	PND12: 22 PND16: 25 (spots)	PND12: intracellular signaling, carbohydrate metabolism, nervous system, oxidative stress, cytoskeleton biogenesis, lipid metabolism PND16: nervous system, intracellular signaling, carbohydrate metabolism, protein metabolism, oxidative stress, cytoskeleton biogenesis, apoptosis	Alexandre-Gouabau et al., 2012
HIP	Pig	Based on birth weight	Post-partum	MS	101	Enriched in females, mild FGR: dopamine signaling, protein complex disassembly, ribosomal structure, protein binding, oxidoreductase activity, GTPase activity, protein folding, metabolism, oxidative stress response Enriched in males, mild FGR: lipid metabolism, fatty acid binding proteins, LDH activity, NAD and GDP binding, cell adhesion, myelin sheath, axon. Enriched severe FGR: neurotransmitter metabolism (cathecolamines), neuronal processes, synaptic plasticity, oxidative phosphorylation, electron transport chain, postsynaptic endosome.	Valent et al., 2019
	Pig	Based on birth weight	385 d post-partum	MS	13	Enriched in normal BW: negative regulation of synaptic vesicle exocytosis, vesicle mediated transport, oxidative phosphorylation, ATP metabolism. Cadherin binding, cytoskeletal protein binding, oxidoreductase activity, RNA binding Enriched in low BW: regulation of mRNA splicing via spliceosome, post-Golgi vesicle-mediated transport. Pre-mRNA binding, cytoskeleton, cadherin binding, actin binding.	Yeste et al., 2022

2-DE: Two-dimensional gel electrophoresis; BW: birth weight; CTX: cortex; FGR: fetal growth restriction; HIP: hippocampus; HYP: hypothalamus; MS: mass spectrometry; PND: postnatal day.

Besides, FGR has been linked to long-term metabolic alterations that may increase the risk of obesity later in life (Mook-Kanamori et al., 2011). In line with this, proteomics on hypothalamic tissue of male rats revealed disruptions in insulin signaling and glucose metabolism, along with disruption of the Krebs cycle. These changes were accompanied by an imbalance in gamma-aminobutyric acid production from glutamate, disruptions in redox homeostasis, and dysfunction of the electron transport chain, leading to reduced adenosine triphosphate production and compromised antioxidant defenses, particularly reflected in changes in glutathione metabolism (Pedroso et al., 2017). This study provides valuable insights into how maternal nutrient restriction may program the hypothalamus to predispose the offspring to obesity. In agreement, another research on the hypothalamus of rats subjected to protein restriction found DEX of proteins involved in neuronal development and key metabolic pathways at PND12, emphasizing alterations in the Krebs cycle and β-oxidation pathway. These alterations became more pronounced at PND16 and were accompanied by increased levels of reactive oxygen species (Alexandre-Gouabau et al., 2012; **Table 4**).

As previously noted, rodents offer a valuable model to investigate the fundamental mechanistic drivers of FGR neuropathology (Swanson and David, 2015). Although the previous analyses do not explicitly justify the selection of the rat as experimental model, their suitability becomes evident when considering the need for longitudinal follow-up to assess obesity and metabolic alterations. Collectively, these analyses demonstrate that nutrient restriction induces metabolic reprogramming of the hypothalamus, resulting in impaired energy production and redox balance, which may ultimately predispose to metabolic disorders.

### Pigs

Studies investigating FGR-related neuropathology in large mammals remain scarce and, the few existing analyses have used pigs, with a focus on hippocampal tissue. A notable study induced FGR by restricting the sows’ feed to 50% of their dietary requirements and delivering the piglets preterm. This study quantified 260 proteins, of which 101 exhibited DEX. As in previous findings, these DEX were linked to oxidative pathways and cytoskeletal components, along with dopamine signaling, ribosomal proteins, protein complexes and translation processes. Interestingly, in mild FGR cases, sex-based differences were detected, suggesting greater tolerance of females to the insult, possibly due to the maintenance of lipid metabolism and antiapoptotic signaling. However, in more severe cases, sex differences were no longer apparent, indicating a threshold where FGR severity surpasses the female adaptive capacity. Apart from the proteins mentioned, others related to G-protein signaling, neurodevelopment, vesicular transport and axon formation contributed to the interaction between sex and protein abundance (Valent et al., 2019; **Table 4**). In short, these DEX confirm that FGR impacts the integrity of the hippocampus detecting groups of proteins already found in human fetal samples, a region critical for learning and memory, potentially contributing to the cognitive impairments associated with this condition, with effects that vary depending on the sex and severity of FGR.

Another study on FGR pigs categorized by birth weight, quantified 2763 proteins in the hippocampus, with only 13 DEX and no sex differences. However, protein abundance differed between low- and normal-birth-weight piglets. In the low-birth-weight group, proteins associated with binding functions, RNA splicing, spliceosome, carbohydrate and lipid metabolism and, mammalian target of rapamycin signaling were enriched. In contrast, oxidative phosphorylation, respiratory chain pathways, cytochrome functions, and catalytic activities were more prominent in the control group (Yeste et al., 2022; **Table 4**). The absence of sex differences could be attributed to the classification method, as relying solely on birth weight may not be enough to accurately identify FGR offspring. The differential expression suggests that low-birth-weight piglets prioritize adaptive regulatory and metabolic pathways to survive under growth restriction, whereas normal-birth-weight piglets maintain robust energy production to support standard growth and development.

Despite being limited, neuroproteomic analyses in pigs have provided insights into the molecular mechanisms underlying FGR neuropathology. The pig is particularly suitable for hippocampal research, as its cytoarchitecture is intermediate between rodents and primates, allowing detailed anatomical and functional characterization. Moreover, pigs exhibit complex cognitive behaviors reliant on hippocampal function (Holm and West, 1994). Because FGR impairs hippocampal-dependent functions such as learning and memory, pigs provide a translationally relevant model for studying hippocampal vulnerability and neurodevelopmental outcomes.

## Limitations

One of the primary limitations of this review steams from the ethical considerations and difficulties in obtaining human tissue for research purposes. Consequently, few proteomic analyses have been conducted to analyze proteomic profiles across different stages of brain development. The very few analyses that have been performed typically involve small sample sizes, and those examining postnatal development encompass a wide range of ages. Additionally, the evolution of neuroproteomic profiles in animal models under physiological conditions remains poorly characterized, especially in large mammals, where no studies have been conducted. Understanding the baseline characteristics of the model organism is essential for accurately interpreting disease mechanisms and pathological responses.

In humans, one major confounding variable is the timing of delivery, as preterm birth is associated with a wide range of physiological alterations that may impact protein expression profiles. The developmental maturity of organs in preterm infants differs significantly from that of term infants, and medical interventions administered to preterm newborns may further influence their proteomic signatures. Additionally, maternal and fetal complications leading to preterm birth could also alter protein levels. A significant number of FGR pregnancies, particularly those involving early-onset FGR (< 32 weeks), require induced preterm delivery to prevent stillbirth. While late-onset FGR can also lead to preterm birth, it is typically indicated closer to term. These variations in delivery timing introduce an additional challenge for the overall interpretation of results.

In animal models, the definition of FGR phenotype varies across analyses, with some relying solely on birth weight as a cutoff, which may be insufficient to distinguish FGR from small for gestational age. When it comes to neuropathology, few proteomic analyses have focused on the brain. In rat models, research has primarily examined brain tissue, without further regional specification and the hypothalamus, while in pigs, the hippocampus has been the region of interest. However, the brain is a highly complex organ with a variety of regions and cell types, each of which may exhibit distinct developmental profiles and respond differently to insults.

Several additional factors may also hinder the comprehensive understanding of results. One issue to consider is the variability introduced during sample collection and handling, as differences in sampling time, protein extraction, and storage methods across analyses may compromise the reproducibility of findings. Furthermore, the use of different technologies, mainly two-dimensional gel electrophoresis and MS could lead to discrepancies in the proteins detected and their respective abundances, further complicating the interpretability of the data. Finally, different analyses employ distinct terms to define the functional characteristics of the proteins identified, with some using Gene Ontology terms and others focusing on biological pathways or a combination of both, while some only provide a list of DEX proteins. The functional analysis sometimes includes proteins altered under treatment conditions. In any case, the functional information presented in this review corresponds to the data highlighted by the authors in their manuscripts.

## Conclusions

The characterization of protein profiles across developmental stages is a valuable tool to describe the biological mechanisms that drive brain development and how these processes are disrupted under pathological conditions. From the earliest phases of neurulation, the transition of pluripotent stem cells into differentiated neural lineages is marked by extensive remodeling of the proteome, involving mechanisms such as cytoskeletal reorganization, metabolic adaptation, and cell cycle regulation. These patterns persist throughout fetal and postnatal development, where regional specificity becomes apparent, revealing the emergence of functionally distinct brain areas.

FGR is a complex and multifactorial condition that presents significant challenges for early detection and effective clinical management. The neurological sequelae associated with FGR arise from a variety of pathogenic mechanisms and functional impairments. Proteomic analysis of body fluids and fetal tissues have shed light on the molecular adaptations contributing to the FGR phenotype, revealing alterations in metabolic processes, oxidative stress-related proteins, immune mediators, coagulation factors, as well as proteins involved in extracellular matrix and cell adhesion. FGR-related injury progresses differently across brain regions, as certain areas show varying vulnerability to hypoxia and hypoglycaemia, and differential responses to these insults. In this context, animal models are essential for identifying critical periods of susceptibility and affected brain regions. The results of these analyses align with human findings, consistently pointing to disruptions in metabolic, oxidative and inflammatory pathways. Insights from such research not only clarify mechanisms of brain damage but also support the identification of region-specific biomarkers. Such an understanding is vital for advancing the development of targeted therapeutic strategies, enabling more effective treatments that specifically address the neurological outcomes while minimizing potential off-target effects.

Additionally, technological advancements have enabled the generation of large-scale proteomic datasets with unprecedented sensitivity and resolution. Looking forward, the integration of multidimensional approaches that combine multi-omic data promises to deepen our understanding of the complex interactions between biological molecules and their functions (Subramanian et al., 2020). The application of artificial intelligence and machine learning further enhances the ability to interpret these complex datasets, reveal hidden patterns, and predict functional outcomes (Adams and Bittremieux, 2025). Together, these strategies offer a powerful framework to advance our understanding of brain development and FGR neuropathology.

## Additional file:

***Additional Table 1:*** *Protein profiles across brain regions and developmental stages.*

## Data Availability

*All relevant data are within the paper and its Additional files.*
